# An Affibody Molecule Is Actively Transported into the Cerebrospinal Fluid via Binding to the Transferrin Receptor

**DOI:** 10.3390/ijms21082999

**Published:** 2020-04-23

**Authors:** Sebastian W. Meister, Linnea C. Hjelm, Melanie Dannemeyer, Hanna Tegel, Hanna Lindberg, Stefan Ståhl, John Löfblom

**Affiliations:** Department of Protein Science, School of Engineering Sciences in Chemistry, Biotechnology and Health, KTH Royal Institute of Technology, AlbaNova University Centre, SE-106 91 Stockholm, Sweden; smeister@kth.se (S.W.M.); lhjelm@kth.se (L.C.H.); melanied@kth.se (M.D.); hannat@biotech.kth.se (H.T.); hanli@kth.se (H.L.); ssta@kth.se (S.S.)

**Keywords:** neurodegenerative disorders, affibody molecules, blood–brain barrier, receptor-mediated transcytosis, transferrin receptor

## Abstract

The use of biotherapeutics for the treatment of diseases of the central nervous system (CNS) is typically impeded by insufficient transport across the blood–brain barrier. Here, we investigate a strategy to potentially increase the uptake into the CNS of an affibody molecule (Z_SYM73_) via binding to the transferrin receptor (TfR). Z_SYM73_ binds monomeric amyloid beta, a peptide involved in Alzheimer’s disease pathogenesis, with subnanomolar affinity. We generated a tri-specific fusion protein by genetically linking a single-chain variable fragment of the TfR-binding antibody 8D3 and an albumin-binding domain to the affibody molecule Z_SYM73_. Simultaneous tri-specific target engagement was confirmed in a biosensor experiment and the affinity for murine TfR was determined to 5 nM. Blockable binding to TfR on endothelial cells was demonstrated using flow cytometry and in a preclinical study we observed increased uptake of the tri-specific fusion protein into the cerebrospinal fluid 24 h after injection.

## 1. Introduction

The blood–brain barrier (BBB) is defined as the structural, physiological, and molecular mechanisms regulating the exchange of molecules between the systemic circulation and the brain [1]. Morphologically, the BBB consists of brain capillary endothelial cells (BCECs) within the microvasculature of the brain [2]. On the abluminal site the BCECs are joined by pericytes and the end-feet of astrocytes [3]. Paracellular passage through the BBB is limited by the presence of tight junction proteins between the individual endothelial cells [4]. Only small hydrophilic molecules can enter the brain via this route [5]. The entry of amphiphilic molecules into the brain is reduced due to expression of efflux transporters in BCECs [6]. While the presence of this tight barrier is essential for central nervous system (CNS) homeostasis, it presents an obstacle for the treatment of diseases of the CNS using biotherapeutics. Only around 0.1%–0.2% of peripherally administered antibodies are typically crossing into the brain [7,8,9]. Potential receptor-mediated transcytosis (RMT) mechanisms of endogenous ligands have been investigated with the aim to increase the brain uptake of biologics in a non-invasive manner. To this end, therapeutic macromolecules have been conjugated to antibodies against receptors or transporters expressed on the BBB. Examples of RMT target proteins include the insulin receptor, low-density lipoprotein receptor-related protein 1, glucose transporter 1, basignin, CD98hc, and the transferrin receptor (TfR) [9]. TfR, which naturally transports iron to the brain [10], is the most widely used target protein for this purpose and has been used in several studies aiming to transport cargo proteins across the BBB [11]. Investigation of the trafficking mechanism using TfR has shed light on the relationship between TfR binding and TfR trafficking [12,13]. In order to achieve optimal brain exposure of the therapeutic cargo, the antibody needs to dissociate from the receptor during or after transcytosis. This can be achieved by utilizing an antibody with moderate to low affinity for TfR [14]. Yu and coworkers could also demonstrate that a high affinity TfR binding antibody is cleared faster from circulation compared to an antibody with lower affinity for TfR [14]. High-affinity TfR binding has been shown to promote degradation of the transferrin receptor in mice [13]. Monovalent binding to the heterodimeric TfR has been shown to induce a more favorable route of intracellular sorting compared to bivalent TfR binding [12,15].

Another possible route for drug delivery into the CNS is via passage over the blood-cerebrospinal fluid (CSF) barrier, which is formed by the epithelium of the choroid plexus [16]. The choroid plexus epithelium expresses TfR and is thought to be involved in iron transport into the brain [17]. CSF communicates freely with brain interstitial fluid through convective flow and diffusion [18]. Systemically administered drugs can reach the brain interstitial fluid by transfer across the BBB, or indirectly by passage over the choroid plexus followed by diffusion/convection transport [19].

In Alzheimer’s disease (AD), the most common form of dementia, an important pathological feature is the deposition of extracellular senile plaques consisting of amyloid beta (Aß) in the brains of patients [20]. Soluble, monomeric Aß peptides self-aggregate into neurotoxic oligomers and insoluble ß-sheet-rich plaques. Aß, in different physical forms, has been the target of numerous active and passive immunotherapeutic campaigns [21]. While several anti-Aß antibodies have proven efficacious in preclinical models [22], most of these antibodies have failed to show sufficient clinical benefits in AD clinical trials [23]. Although antibodies that are specific for the monomeric form of Aß have not yet demonstrated better efficacy than agents targeting for example the amyloid plaques, it has been suggested that they might still have a higher potential. The motivation is mainly that potential future therapies probably need to start at the pre-symptomatic stage of the disease and thus should target Aß species that are found early in the disease progression [24]. We have recently reported on the generation of an affibody molecule that binds monomeric Aß with 60 pM affinity. Affibody molecules are alternative scaffold proteins that are engineered by directed evolution (e.g., phage display technology). We have previously developed an affibody-based Aβ-binder by phage display technology. In a subsequent affinity-maturation effort, we isolated a high-affinity variant (denoted Z_SYM73_) from second-generation affibody libraries displayed on bacteria [25].

Z_SYM73_ has an unusual structure and forms a disulfide-stabilized heterodimer in complex with the Aß peptide. Upon binding, both the affibody and the Aß peptide fold, forming a beta-sheet with Aß in a beta-hairpin conformation [26]. When bound, the aggregation-prone parts of Aß are buried in a tunnel-like cavity and the affibody efficiently inhibits the aggregation. In an APP/PS1 double transgenic mouse study, treatment with Z_SYM73_ led to prevention of the amyloid burden build up in both cortex and hippocampus as well as prevention of decline in cognitive function [27].

Encouraged by these positive results, the aim here was to investigate strategies that might be employed in future therapeutic studies to increase brain exposure of Z_SYM73_, which has the potential to also improve efficacy. It has previously been demonstrated that the CNS uptake of rat 8D3 mAb against mouse TfR is substantially higher than for non-TfR binding antibodies [28,29,30]. We genetically fused Z_SYM73_ to a single-chain variable fragment (scFv) of the 8D3 mAb and an engineered albumin-binding domain (ABD) [31], which was included also in the preclinical study with Z_SYM73_ [27]. The engineered ABD and its binding to serum albumin has been shown in a number of both preclinical and clinical studies to prolong the circulatory half-life of fusion proteins by decreasing renal filtration and indirect recycling via the neonatal Fc receptor [32,33,34,35]. The tri-specific fusion protein thus comprised (i) a monovalent TfR-binding scFv, (ii) the therapeutic candidate Z_SYM73_ with affinity for monomeric Aß, and (iii) the ABD for prolonged in vivo circulation, at less than a third of the molecular weight of a standard IgG mAb (44 kDa compared to 150 kDa).

## 2. Results

### 2.1. Design and Production of scFv8D3-Z_SYM73_-ABD and Z_SYM73_-ABD

**Figure 1:**
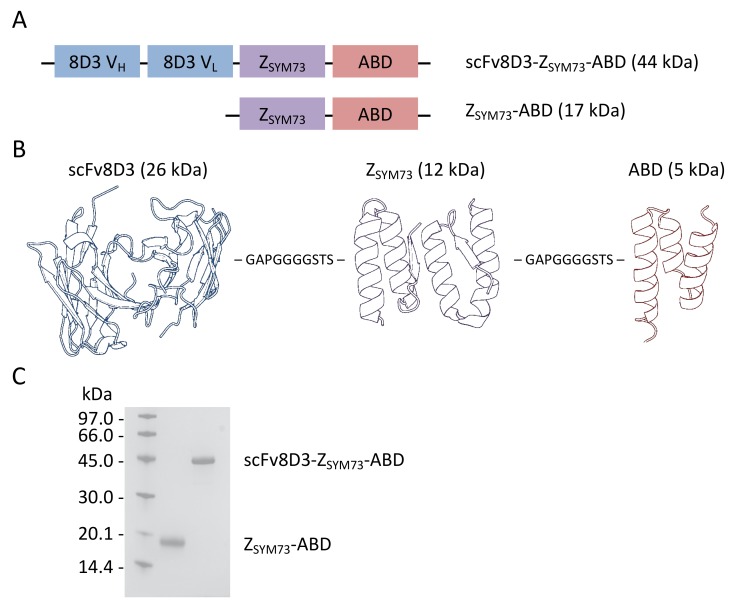
(**A**) Schematic representation of the tri-specific scFv8D3-Z_SYM73_-ABD and the control protein Z_SYM73_-ABD. Abbreviations scFv = single-chain variable fragment, Z = affibody, ABD = albumin-binding domain. (**B**) Schematic picture over the structure of scFv8D3-Z_SYM73_-ABD with linkers between the subunits as amino acid sequences. The schematic structure is composed of following PDB IDs: scFv: 1KTR Zsym: 2OTK and ABD: 1GJT. (**C**) SDS-PAGE analysis of purified proteins. Purified scFv8D3-Z_SYM73_-ABD and Z_SYM73_-ABD appear as a single band of the correct size. The original and non-cropped gel is shown in Supplementary Figure S1.

Z_SYM73_ is a heterodimeric affibody with high and specific affinity for the monomeric amyloid beta peptide (Figure 1A,B). The two subunits are connected by a flexible glycine/serine linker. For extending the in vivo half-life, we fused the affibody to a small albumin-binding domain (ABD; 46 amino acids) that is originally derived from streptococcal protein G [36] (Figure 1A,B). The albumin-binding domain used in this study (ABD_035_ [21]; denoted only ABD hereinafter) has been engineered to femtomolar affinity for human serum albumin by directed evolution [21]. It also binds to serum albumin from other species, including mouse, and has successfully been used to extend the circulatory half-life of affibody fusion proteins in both preclinical and clinical studies [37,38]. Z_SYM73_-ABD was included in this study to compare CSF uptake in absence of TfR-binding (Figure 1A,B).

To investigate the potential of transferrin-receptor (TfR) targeting for increasing the uptake of Z_SYM73_ in the central nervous system (CNS), we used the previously investigated TfR-specific antibody 8D3 [39]. The rat 8D3 mAb is specific for mouse TfR [40] and was reformatted into a synthetic scFv in the heavy (V_H_) to light (V_L_) chain orientation, separated by a 16 amino-acid flexible linker (^N^GTTAASGSSGGSSSGA^C^) [39]. The resulting scFv8D3 was then fused to the N-terminus of Z_SYM73_-ABD [17] via a 10 amino-acid linker (^N^GAPGGGGSTS^C^).

ScFv8D3-Z_SYM73_-ABD and Z_SYM73_-ABD were produced in CHO cells and purified using affinity chromatography with human serum albumin (HSA) immobilized as ligand on a sepharose matrix, and followed by endotoxin removal by purification on EndoTrap columns. Both fusion proteins were observed as single bands of correct size after SDS-PAGE, demonstrating high sample purity (Figure 1C, the non-cropped gel is shown in Supplementary Figure S1). Quantification of the recovered proteins via amino acid analysis was carried out to determine protein concentrations. Production yields were approximately 35 mg of purified protein per liter cell culture medium for both proteins.

### 2.2. SPR Assays for Analysis of the Interaction between Recombinant Mouse TfR, MSA, and Aß_1-40_ with the Trispecific Fusion Protein

**Figure 2:**
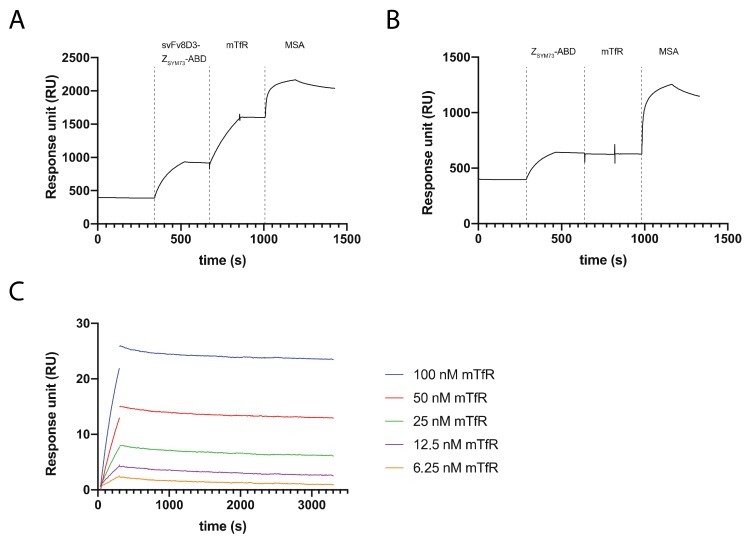
Surface plasmon resonance (SPR)-based biosensor assays. (**A**) Representative sensorgram showing the results from a triple co-inject assay. Aβ_1-40_ is immobilized on the surface, and scFv8D3-Z_SYM73_-ABD, mTfR, and mouse serum albumin (MSA) are injected subsequently. (**B**) Representative sensorgram showing the results from a triple co-inject assay. Aβ_1-40_ is immobilized on the surface, and Z_SYM73_-ABD, mTfR, and MSA are injected subsequently. Respective start points of injections are marked with a dashed line and the injected protein is stated over respective part of the sensorgram. (**C**) Representative sensorgram from the kinetic analysis of the interaction between mTfR and scFv8D3-Z_SYM73_-ABD. All experiments were performed in duplicates. The gaps in the sensorgrams at the end of injection are from removal of spikes in signal by the Biacore evaluation software.

First, we performed an in vitro binding analysis to evaluate the retained functionality of each of the domains (scFv8D3, Z_SYM73_, and ABD) in the designed fusion protein using SPR-based biosensor assays. ScFv8D3-Z_SYM73_-ABD or Z_SYM73_-ABD were injected over a streptavidin surface on which biotinylated Aβ_1-40_ was first indirectly immobilized. Mouse TfR and mouse serum albumin (MSA) were subsequently injected over the surface without regeneration. The results showed that scFv8D3-Z_SYM73_-ABD is capable of simultaneously binding to Aß_1-40_, TfR, and MSA (Figure 2A), demonstrating that both the N-terminus and C-terminus of Z_SYM73_ tolerate conjugation to fusion partners. As expected, no binding of Z_SYM73_-ABD to TfR could be observed (Figure 2B).

Next, the affinity of the interaction between scFv8D3-Z_SYM73_-ABD and mouse TfR was investigated. In nature, TfR is a homodimeric receptor [41] and the two domains are covalently connected by two disulfide bonds between Cys 89 and Cys 98 in the respective domain, which can lead to avidity effects when interacting with surface-captured scFv8D3-Z_SYM73_-ABD. The recombinant extracellular domain of TfR that was used in SPR starts at Cys 89 and is then most likely forming the two disulfide bonds and consequently has potential for dimerization. In order to assess the dimeric status of the receptor, we analyzed dithiothreitol (DTT)-treated TfR and non-treated TfR using SDS-PAGE. The results demonstrated that a relatively large proportion of the receptor is in a multimeric form (Supplementary Figure S2). Hence, to control for avidity effects in the kinetic analysis, we captured scFv8D3-Z_SYM73_-ABD on HSA surfaces at three different immobilization levels. The mean equilibrium dissociation constant for the kinetic analysis was determined to be 5 nM. We only observed a small increase in off-rates for lower capture levels indicating small avidity effects (Table 1). Representative SPR measurement for the kinetic analysis of the interaction between mouse TfR and scFv8D3-Z_SYM73_-ABD are shown in Figure 2C.

### 2.3. Flow Cytometry for Analysis of the Interaction between the Trispecific Fusion Protein and Mouse TfR-Expressing Endothelial Cells

**Figure 3:**
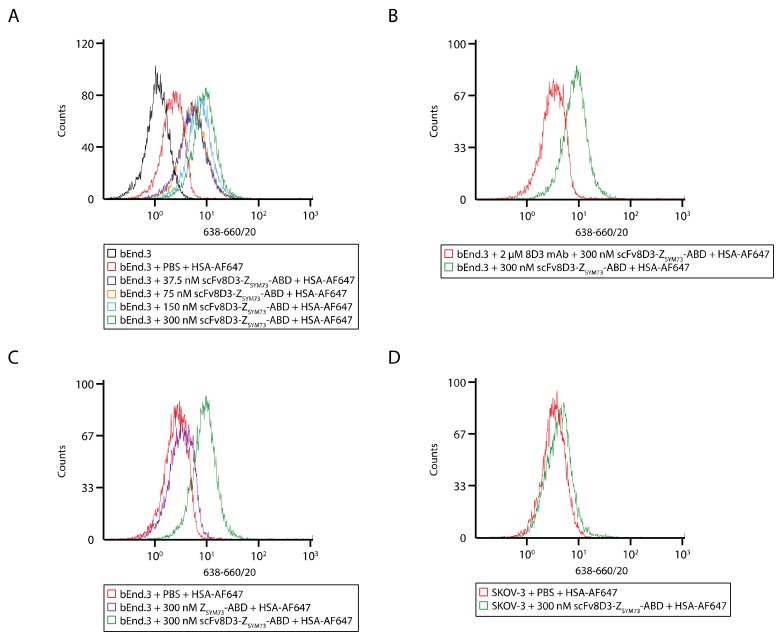
Flow-cytometric analysis of scFv8D3-Z_SYM73_-ABD binding to cells. (**A**) Representative histograms showing results from the flow-cytometric analysis of bEnd.3 cells treated with 37.5 nM, 75 nM, 150 nM, and 300 nM scFv8D3-Z_SYM73_-ABD. As a control, cells labeled only with HSA-AF647 and unlabeled cells were analyzed. (**B**) Representative histograms showing results from a blocking experiment using the parental mAb 8D3. (**C**) Representative histograms showing results from a control experiment using Z_SYM73_-ABD. The MFI for cells treated with scFv8D3-Z_SYM73_-ABD was 8.8 ± 0.2 and the MFI for cells treated with Z_SYM73_-ABD was 3.7 ± 0.5. An unpaired *t* test demonstrated significant difference (*p* value = 0.0059). (**D**) Representative histograms showing results from the flow-cytometric analysis of SKOV-3 cells treated with 300 nM scFv8D3-Z_SYM73_-ABD. As a control, cells labeled only with HSA-AF647 were analyzed. The wavelength of the excitation laser and bandwidth of the fluorescence detection filter is shown on the *x*-axis label in nm.

Flow cytometry was used to assess whether scFv8D3-Z_SYM73_-ABD could bind TfR in a cellular context. Mouse brain endothelial cells (bEnd.3) were treated with 37.5 nM, 75 nM, 150 nM, and 300 nM scFv8D3-Z_SYM73_-ABD as well as PBS as control, and HSA labeled with Alexa Fluor 647 (HSA-AF647) was used as a secondary reagent. A concentration-dependent shift in fluorescence signal was observed for cells treated with scFv8D3-Z_SYM73_-ABD compared to the PBS control, confirming binding to TfR in a cellular context (Figure 3A and Supplementary Figure S3B). Since fluorescently labeled HSA was used as secondary reagent, the results also confirmed simultaneous binding to albumin and TfR. Co-incubation of bEnd.3 cells with scFv8D3-Z_SYM73_-ABD and a 6.6-fold molar excess of unlabeled parental monoclonal antibody 8D3 resulted in decrease in signal, indicating specific binding to TfR (Figure 3B). We did not observe binding of the control protein Z_SYM73_-ABD to bEnd.3 cells or binding of scFv8D3-Z_SYM73_-ABD to the human cell line SKOV-3 (murine TfR negative) (Figure 3C,D).

### 2.4. Bioavailability of the Affibody Fusion Proteins in Mouse CSF

The CSF bioavailability of scFv8D3-Z_SYM73_-ABD and Z_SYM73_-ABD was investigated in a mouse study. Male NMRI mice received a single 87.8 nmol/kg intravenous dose of either scFv8D3-Z_SYM73_-ABD or Z_SYM73_-ABD. One animal died directly after the injected dose, due to an air bubble in the syringe. CSF and serum samples were obtained from mice terminated after 3 h, 24 h, and 48 h. The specific concentrations of the two proteins in the biological samples were determined in an ELISA (Supplementary Table S1).

**Figure 4:**
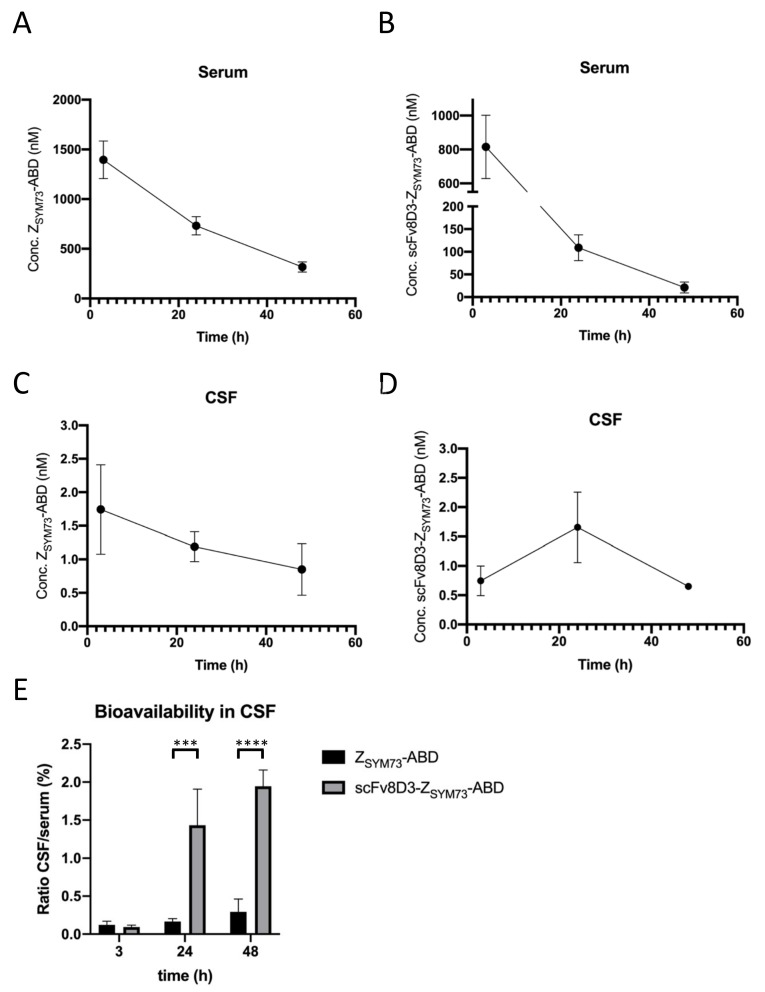
Concentrations of Z_SYM73_-ABD and scFv8D3-Z_SYM73_-ABD in NMRI mouse serum and CSF at 3 h, 24 h, and 48 h post administration. (**A**) Pharmacokinetic profile of Z_SYM73_-ABD in mouse serum. (**B**) Pharmacokinetic profile of scFv8D3-Z_SYM73_-ABD in mouse serum. (**C**) Pharmacokinetic profile of Z_SYM73_-ABD in mouse cerebrospinal fluid (CSF). (**D**) Pharmacokinetic profile of scFv8D3-Z_SYM73_-ABD in mouse CSF. (**E**) Bioavailability in CSF, expressed as CSF-to-serum ratio, of Z_SYM73_-ABD and scFv8D3-Z_SYM73_-ABD at 3 h, 24 h, and 48 h post administration. Unpaired *t* tests on data from 24 and 48 h demonstrated significant differences between CSF/serum ratios for Z_SYM73_-ABD and scFv8D3-Z_SYM73_-ABD (*** *p* value ≤ 0.001, **** *p* value ≤ 0.0001).

We first evaluated the pharmacokinetic profile of scFv8D3-Z_SYM73_-ABD and Z_SYM73_-ABD in serum over the time-course of 48 h (Figure 4A,B). By fitting the data using a one phase decay model, we estimated a serum half-life of around 26 h for Z_SYM73_-ABD, which is in accordance with the 28.8 h serum half-life of MSA in mice [42]. We observed a faster serum clearance for scFv8D3-Z_SYM73_-ABD, resulting in an estimated serum half-life of around 7 h.

Next, we determined the absolute concentrations of the two proteins in CSF at 3 h, 24 h, and 48 h. Some of the CSF samples had to be excluded from the analysis due to contamination with blood or protein concentrations below the ELISA’s sensitivity (Supplementary Table S1). The CSF concentrations of Z_SYM73_-ABD steadily declined over the observed time course, with concentrations of 1.74 nM, 1.19 nM, and 0.85 nM at 3 h, 24 h, and 48 h, respectively (Figure 4C). The CSF concentration of scFv8D3-Z_SYM73_-ABD doubled from 0.75 nM to 1.66 nM between 3 h and 24 h post injection (Figure 4D). We determined a mean concentration of 0.65 nM scFv8D3-Z_SYM73_-ABD in the CSF samples after 48 h (Figure 4D).

Based on this data, we determined CSF bioavailability, expressed as CSF-to-serum ratios, of the two proteins over 48 h. We observed a steep increase in CSF bioavailability of scFv8D3-Z_SYM73_-ABD between 3 h and 24 h, with CSF-to-serum ratios of 0.09% and 1.43%, respectively (Figure 4E). At 48 h post injection, the CSF-to-serum ratio of scFv8D3-Z_SYM73_-ABD was 1.94%. The CSF bioavailability of the control protein Z_SYM73_-ABD was 0.12%, 0.16%, and 0.29% at 3 h, 24 h, and 48 h, respectively (Figure 4E). The CSF bioavailability of Z_SYM73_-ABD is in accordance with a recent study carried out in rats [27] and reflects values reported for passive protein uptake into the CNS [43]. The fusion of scFv8D3 to Z_SYM73_-ABD led to an 9-fold increase in CSF bioavailability after 24 h indicating an active transport mechanism into CSF.

## 3. Discussion

In this present study we explored a strategy that could potentially increase the brain uptake of an affibody molecule via transferrin receptor-mediated transcytosis in the future. Engagement of the TfR has successfully been used in previous studies to transport cargo proteins across the BBB [14,28,44].

Here, we designed a tri-specific fusion protein consisting of a single-chain variable fragment (scFv) of the mouse TfR-specific antibody 8D3, the Aβ-specific affibody molecule Z_SYM73_, and an engineered albumin-binding domain (ABD) (Figure 1A,B). There is a risk that fusion to ABD and scFv might affect the interaction with Aβ. In an SPR assay, we demonstrated that scFv8D3-Z_SYM73_-ABD was able to simultaneously engage with Aβ_1-40_, mouse TfR, and MSA, confirming the tri-specific nature of the fusion protein. We have also previously reported on the therapeutic effect of Z_SYM73_-ABD in a murine AD model with promising results, and since ABD is cross reactive to MSA, this is further indication that albumin-binding has no dramatic negative effect on target binding. We report an affinity (K_D_) of scFv8D3-Z_SYM73_-ABD for mouse TfR of 5 nM (Figure 2C and Table 1). The observed affinity is about 3-fold stronger than previously reported monomeric 8D3 affinity, as determined by ELISA [28]. When investigating the relationship between TfR affinity and brain uptake of TfR antibodies, Yu et al. reported highest brain exposure for antibodies with an affinity for TfR of around 50 nM–100 nM [14]. More recently, apparent TfR affinities of 0.6 nM and 8 nM led to increased brain uptake of antibody variants [28,44]. Our engineered fusion protein engages with TfR in a monovalent binding mode, which has been shown to be important for transport across the BBB [12]. We next demonstrated blockable binding of scFv8D3-Z_SYM73_-ABD to TfR on mouse brain endothelial cells (bEnd.3).

The CSF uptake and serum pharmacokinetics of scFv8D3-Z_SYM73_-ABD and the control protein Z_SYM73_-ABD were assessed in NMRI mice. It has recently been shown that Z_SYM73_-ABD treatment leads to prevention of the amyloid burden build up in both cortex and hippocampus as well as prevention of cognitive function decline in APP/PS1 double transgenic mice. The brain bioavailability of Z_SYM73_-ABD in mice is however unknown [27].

We decided to assess protein uptake into CSF as a surrogate for brain uptake. It has been reported that drug concentrations in CSF can be used for estimating the concentration in brain interstitial fluid [45,46]. We reasoned that affibody concentrations in CSF do not take into account proteins bound to TfR on the BBB and hence reflect active, free affibody concentrations in CSF. Wang and coworkers concluded that CSF-to-serum ratios are indicators of antibody CNS uptake after the antibody concentration in CSF reached a maximum [43].

In contrast to the results for Z_SYM73_-ABD, we observed a steep increase in CSF concentration of the scFv8D3-Z_SYM73_-ABD between 3 and 24 h post injection, indicating active receptor-mediated uptake (Figure 4D). It should be noted that the concentration of the trispecific protein in CSF was approximately 2-fold lower at 3 h p.i., and only moderately higher (1.4-fold) at 24 h p.i. compared to the control protein. Clearance from serum was drastically faster for scFv8D3-Z_SYM73_-ABD compared to the control protein Z_SYM73_-ABD, with estimated circulatory serum half-lives of 7 h and 26 h, respectively (Figure 4A,B). Fast serum clearance has been reported for TfR antibodies and is likely due to binding to TfR expressed in peripheral tissues such as liver and kidney [44]. The CSF bioavailability, expressed as the ratio of CSF-to-serum concentrations, for Z_SYM73_-ABD was 0.12%, 0.16%, and 0.29% at 3 h, 24 h, and 48 h, respectively. We observed a significant increase in CSF bioavailability for scFv8D3-Z_SYM73_-ABD, with ratios of 1.43% and 1.94% at 24 h and 48 h, respectively. Brain-to-serum concentration ratios between 0.91% to 2.11% at 24 h post injection have been reported by Yu and coworkers for monovalent TfR antibodies following injection of a similar dose (20 mg/kg or approximately 130 nmol/kg) [14], indicating that protein concentrations in CSF might be used as a surrogate for brain protein concentrations.

In this study, we investigated CSF bioavailability of affibody fusion proteins after administrating a therapeutically relevant dose of 87.8 nmol/kg. These doses are comparable to doses administered in a preclinical study of Z_SYM73_-ABD and significantly larger than doses used in previous studies exploiting TfR brain uptake using the 8D3 antibody [27,28,44].

The increase in CSF bioavailability could be the result of either active transport over BBB, followed by diffusion transport from the brain interstitial fluid into CSF or by passage over the epithelium of the choroid plexus [16]. However, uptake at the choroid plexus has been demonstrated to inversely correlate with the molecular weight of compounds [47]. Since the bioavailability of the larger scFv8D3-Z_SYM73_-ABD (44 kDa) is higher compared with the smaller Z_SYM73_-ABD (17 kDa), this indicates that the uptake into CSF is not primarily driven by passive passage over the epithelium of the choroid plexus. Still, using CSF as a surrogate for estimating uptake into brain parenchyma is controversial and should be considered as indications [47]. Future studies on target engagement and therapeutic effect will hopefully contribute to the understanding of the mechanisms. Another option is to measure the concentration of the affibody fusion proteins using ELISA on brain homogenate. In such studies, it is important to note that measurements on brain homogenates typically do not distinguish between proteins that have passed over the endothelium layer into the brain parenchyma, and the fraction of proteins still associated with the endothelial cells, and more sophisticated methods (e.g., capillary depletion methods [48]) should hence be used to avoid overestimating the uptake [49].

Although the results indicate active uptake into CSF that is mediated by binding to TfR, the much faster blood clearance of the scFv8D3-Z_SYM73_-ABD is far from optimal. In future studies, it would be interesting to investigate both the brain uptake and the pharmacokinetics of the scFv8D3-Z_SYM73_-ABD at different doses. Due to the fast serum clearance of the protein, higher doses might be necessary to achieve a therapeutic relevant concentration in CSF, but this might impede the future clinical utility. Moreover, we would like to explore lower affinity binding domains for TfR and different valences of 8D3, which could influence both uptake into CNS as well as the blood clearance rate. Dissociation of the brain shuttle molecules from TfR expressed on the BBB might be slow, leading to lower active concentration in brain and consequently lower amounts of protein detectable in CSF. This could be achieved by mutating scFv8D3 and selection of lower affinity variants or by replacing the antibody fragment for an affibody molecule with moderate affinity towards TfR. It should also be noted that the 8D3 antibody is not cross-reactive to human TfR and was only used in this study to explore the potential of TfR-targeting for increasing uptake into CNS. If TfR-specific affibody molecules were to be developed in the future, achieving cross-reactivity between human TfR and corresponding TfRs in model animals should be a focus.

At 44 kDa, the scFv8D3-Z_SYM73_-ABD presented in this study is significantly smaller than comparable therapeutic bispecific antibodies. It should be noted that the drug complex would be substantially larger in blood when associated with albumin, which will affect parameters such as diffusion and penetration in tissues. There is still a potential positive effect of small size in terms of possibilities for higher molar concentrations in formulations which could open up for alternative routes of administration (e.g., subcutaneous injections) in the future. Moreover, since albumin concentration is lower in the brain, the fraction of non-HSA associated drug might be higher in this compartment, which could have positive effects on brain biodistribution. Moreover, CSF is only an indication of increased uptake in the brain. Target engagement by measuring therapeutic effect is an important next step to obtain more experimental evidence on brain uptake and follow-up efforts will be focused on such studies.

## 4. Materials and Methods

### 4.1. Design and Molecular Cloning of Affibody Fusion Proteins

The rat 8D3 mAb against mouse TfR was reformatted into a synthetic scFv in the heavy (V_H_) to light (V_L_) chain orientation, separated by a 16 amino-acid flexible linker (^N^GTTAASGSSGGSSSGA^C^). The 8D3 scFv was fused to the N-terminus of Z_SYM73_-ABD [27] via a 10 amino-acid linker (^N^GAPGGGGSTS^C^). The albumin-binding domain used in this study (ABD_035_ [21]; denoted only ABD hereinafter) has been engineered to femtomolar affinity for human serum albumin by directed evolution [21]. The resulting fusion protein is hereafter denoted as scFv8D3-Z_SYM73_-ABD. The gene encoding scFv8D3-Z_SYM73_-ABD was ordered from Genewiz (GENEWIZ Germany GmbH, Leipzig, Germany). The genes encoding scFv8D3-Z_SYM73_-ABD and Z_SYM73_-ABD were inserted into pQMCF1 (Icosagen Cell Factory OU, Tartu, Estonia) [50]. The genes encoding scFv8D3-Z_SYM73_-ABD and Z_SYM73_-ABD were amplified by polymerase chain reaction (PCR) using Q5 high-fidelity polymerase (New England Biolabs, Ipswich, MA, USA). Specific primers were used to introduce a *Not*I sequence upstream-, and an *Asc*I sequence downstream of the respective gene. The pQMCF1 vector (Icosagen Cell Factory OU, Tartu, Estonia) and the two PCR products were digested using *Not*I-HF and *Asc*I restriction enzymes (NEB). The digested vector and inserts were purified using QIAquick Gel Extraction Kit (Qiagen GmbH, Hilden, Germany) and QIAquick PCR Purification Kit (Qiagen), respectively. The purified vector and inserts were subsequently ligated using T4 DNA Ligase (NEB). By utilizing the *Not*I and *Asc*I restriction sites the genes were ligated in fusion with a N-terminal CD33 secretion signal peptide present in the vector. The pQMCF1 plasmid moreover contains a CMV promoter [50]. The two plasmids (pQMCF1 scFv8D3-ZSYM73-ABD and pQMCF1 ZSYM73-ABD) were transformed into chemically competent TOP10 *Escherichia coli* cells by heat shock (Thermo Fisher Scientific, Waltham, MA, USA). Plasmids were prepared using QIAprep Spin Miniprep Kit (Qiagen GmbH, Hilden, Germany) and the sequences were verified by Sanger DNA sequencing (Microsynth AG, Balgach, Switzerland).

### 4.2. Protein Expression, Purification, and Quality Control

ScFv8D3-Z_SYM73_-ABD and Z_SYM73_-ABD were expressed in Chinese hamster ovary (CHO) EBNALT 85 cells using the Icosagen QMCF technology (an episomal protein expression system, which uses mammalian cells that are genetically modified and designed plasmids). After 12 days of cultivation, cell culture supernatants were spiked with 10× tris-buffered saline (TST) to a final concentration of 1× TST. ScFv8D3-Z_SYM73_-ABD and Z_SYM73_-ABD were recovered from the cell culture supernatant using affinity chromatography with human serum albumin (HSA) as a ligand immobilized to sepharose matrix, as described elsewhere [51]. The recovered proteins were buffer-exchanged to phosphate-buffered saline (PBS) using PD-10 desalting columns (GE Healthcare Life Sciences, Uppsala, Sweden). Endotoxin removal was carried out using EndoTrap^®^ red columns (Lionex GmbH, Braunschweig, Germany) according to the manufacturer’s instructions. The purity of the recovered proteins was evaluated by SDS-PAGE. Quantification of the proteins was carried out by amino acid analysis (Alphalyse A/S, Odense, Denmark). All proteins were stored in PBS at −80 °C.

### 4.3. Analysis of Binding to Recombinant Proteins Using SPR-Based Biosensor Assays

All surface plasmon resonance (SPR) experiments were performed on a Biacore T200 (GE Healthcare Life Sciences, Uppsala, Sweden) with PBS containing 0.5% Tween 20 as running buffer. Firstly, trispecific simultaneous binding of the affibody fusion proteins was analyzed. Biotinylated amyloid beta 1-40 (AnaSpec, Fremont, CA, USA) was captured on a streptavidin sensor chip (GE Healthcare Life Sciences, Uppsala, Sweden) according the manufacturer’s recommendations to a capture level of 130 RU. Three hundred nanomoles of either scFv8D3-Z_SYM73_-ABD or Z_SYM73_-ABD was initially injected over the surface for 180 s at a flow rate of 30 μL/min. After a dissociation phase of approximately 150 s, 300 nM of recombinant his-tagged mouse TfR (Sino Biological Inc., Beijing, China) was injected over the surface for 180 s. Following another dissociation phase of approximately 150 s, 300 nM mouse serum albumin (Merck KGaA, Darmstadt, Germany) was injected for 180 s.

Kinetic data on the scFv8D3 interaction with mouse TfR was obtained by capturing scFv8D3-Z_SYM73_-ABD on human serum albumin (HSA) immobilized on the surface, followed by injections of various concentrations of mouse TfR. To this end, albumin from human serum (Merck KGaA, Darmstadt, Germany) was immobilized on three different surfaces of a CM5 sensor chip (GE Healthcare Life Sciences, Uppsala, Sweden) according to the manufacturer’s protocol. Immobilization levels of HSA reached 747 response units (RU), 487 RU, and 229 RU for the three different surfaces. The flow rate for all following injections was 30 μL/min. 80 nM scFv8D3-Z_SYM73_-ABD was injected over all surfaces for 10 s, followed by 300 s injections of mouse TfR (Sino Biological Inc., Beijing, China) in concentrations of 100 nM, 50 nM, 25 nM, 12.5 nM, 6.25 nM, and 0 nM. Dissociation was monitored by injecting running buffer for 3000 s. The surfaces were regenerated by injecting 10 mM HCl for 30 s. All SPR experiments were carried out in duplicates.

### 4.4. Analysis of Binding to TfR on Mouse Brain Endothelial Cells Using Flow Cytometry

The monoclonal anti-mouse TfR antibody 8D3 was purchased from Novus Biologicals (Novus Biologicals LLC, Centennial, CO, USA). The bEnd.3 mouse brain endothelial cell line (ATCC) was cultured in Dulbecco’s Modified Eagle’s Medium (Merck KGaA) complemented with 10% fetal bovine serum. The SKOV-3 cell line (ATCC) was cultured according to the manufacturer’s protocol. At approximately 80% confluency, cells were harvested using TrypLE^TM^ Express (ThermoFisher Scientific, Waltham, MA, USA) according to the manufacturer’s protocol. Around 100,000 cells were resuspended in the respective protein solution in PBS + 1% bovine serum albumin (BSA; PBSB) and incubated for 45 min at 4 °C under constant agitation. The supernatant was discarded by centrifugation at 1700 rpm and 4 °C for 4 min. Cells were subsequently resuspended in 100 nM HSA-Alexa Fluor 647 conjugate (produced in-house) in PBSB and incubated for 20 min at 4 °C under constant agitation. The supernatant was discarded as described above and the cells were resuspended in 400 uL ice-cold PBSB. The cells were analyzed using a Gallios^TM^ flow cytometer (Beckman Coulter Inc., Indianapolis, IN, USA). Gates based on forward and side scatter intensities were used to analyze intact and single cells (Supplementary Figure S3A). All flow cytometry experiments were carried out in duplicates on separate biological samples.

### 4.5. Analysis of CNS Uptake in a Mouse Model

The animal study was carried out by Adlego Biomedical AB, Solna, Sweden. Ethics permit No. 4570-2019 approved by the regional animal experimental committee in Stockholm (North). 36 male NMRI mice were divided into two groups. Twenty-one mice received an 87.8 nmol/kg dose of Z_SYM73_-ABD and 15 mice received an 87.8 nmol/kg dose of scFv8D3-Z_SYM73_-ABD via intravenous injection in a lateral tail vein. Subgroups of 7 (Z_SYM73_-ABD) or 5 (scFv8D3-Z_SYM73_-ABD) mice were terminated at 3, 24, and 48 h after administration. Blood samples were collected from the orbital plexus and CSF samples were collected from the cisterna magna at termination. Serum was extracted from the blood samples and all sampled were stored at −20 °C within 1 h of collection. Samples were thawed on ice and measured in an ELISA including a freshly prepared calibration standard (reference material from identical batch). Briefly, a 96-well plate was coated with mouse monoclonal anti-affibody antibodies (Affibody AB, Solna, Sweden). Serum received from one untreated mouse was used to prepare a 1% (*v*/*v*) solution in Blocker^TM^ Casein in PBS (ThermoFisher Scientific, Waltham, MA, USA). All serum samples and serum standards were diluted in the 1% (*v*/*v*) serum solution. For dilution of the CSF samples and CSF standards, a 1% (*v*/*v*) solution of CSF received from one untreated rat in Blocker^TM^ Casein (ThermoFisher Scientific, Waltham, MA, USA) was prepared. The samples and calibration standards were diluted and added to the wells. Polyclonal rabbit anti-ABD antibodies (Affibody AB) were added, followed by horseradish peroxidase (HRP)-conjugated polyclonal donkey-anti-rabbit-IgG antibodies (Jackson Immunoresearch, West Grove, PA, USA). Detection was carried out by addition of TMB substrate (ThermoFisher Scientific, Waltham, MA, USA) and the reaction was stopped using 2 M sulfuric acid. Independent experiments (*N*) were performed on individual animals. Parallel ELISA experiments (*n*) were performed in triplicate on separate biological samples and calibration standards in parallel on the same day.

## Figures and Tables

**Table 1:** Results from the kinetic analysis of the interaction between mTfR and scFv8D3-Z_SYM73_-ABD using SPR. ScFv8D3-Z_SYM73_-ABD was captured on human serum albumin (HSA) surfaces at three different levels and mTfR was injected over the surfaces. Mean values of two separate experiments and standard deviations are shown.

Immobilization Level	R_max_ (RU)	k_a_ (1/Ms)	k_d_ (1/s)	K_D_ (nM)
Surface 1: High (747 RU)	73.3 ± 9.8	1.4 ± 0.1 × 10^4^	3.5 ± 0.1 × 10^−5^	2.3 ± 0.02
Surface 2: Intermediate (487 RU)	61.3 ± 19.4	1.2 ± 0.3 × 10^4^	7.6 ± 0.9 × 10^−5^	6.6 ± 1.2
Surface 3: Low (229 RU)	21.9 ± 4.2	2.0 ± 0.3 × 10^4^	9.9 ± 0.5 × 10^−5^	5.2 ± 0.6

R_max_, maximal capacity of the sensor chip surface; k_a_, association rate constant; k_d_, dissociation rate constant; K_D_, equilibrium constant.

## Supplementary Materials

The following are available online at https://www.mdpi.com/1422-0067/21/8/2999/s1, Figure S1: Non-cropped SDS-PAGE analysis of purified proteins, Figure S2: SDS-PAGE analysis of reduced- and non-reduced recombinant proteins, Figure S3: Representative plot from flow-cytometric analysis with gating indicated, Table S1: Serum and CSF concentrations, and C_CSF_/C_serum_ ratios of the two investigated proteins in individual mice.

## References

[1] Saunders N.R., Habgood M.D., Møllgård K., Dziegielewska K.M. (2016). The biological significance of brain barrier mechanisms: Help or hindrance in drug delivery to the central nervous system?. F1000Research.

[2] Thomsen M.S., Routhe L.J., Moos T. (2017). The vascular basement membrane in the healthy and pathological brain. J. Cereb. Blood Flow. Metab..

[3] Abbott N.J., Rönnbäck L., Hansson E. (2006). Astrocyte–endothelial interactions at the blood–brain barrier. Nat. Rev. Neurosci..

[4] Brightman M.W., Reese T.S. (1969). Junctions between intimately apposed cell membranes in the vertebrate brain. J. Cell Biol..

[5] Hawkins B.T., Davis T.P. (2005). The Blood-Brain Barrier/Neurovascular Unit in Health and Disease. Pharmacol. Rev..

[6] Abbott N.J., Patabendige A.A.K., Dolman D.E.M., Yusof S.R., Begley D.J. (2010). Neurobiology of Disease Structure and function of the blood—brain barrier. Neurobiol. Dis..

[7] Poduslo J.F., Curran G.L., Berg C.T. (1994). Macromolecular permeability across the blood-nerve and blood-brain barriers. Proc. Natl. Acad. Sci. USA.

[8] Yu Y.J., Watts R.J. (2013). Developing Therapeutic Antibodies for Neurodegenerative Disease. Neurotherapeutics.

[9] Zuchero Y.J.Y., Chen X., Bien-Ly N., Bumbaca D., Tong R.K., Gao X., Zhang S., Hoyte K., Luk W., Huntley M.A. (2016). Discovery of Novel Blood-Brain Barrier Targets to Enhance Brain Uptake of Therapeutic Antibodies Article Discovery of Novel Blood-Brain Barrier Targets to Enhance Brain Uptake of Therapeutic Antibodies. Neuron.

[10] Duck K.A., Connor J.R. (2016). Iron uptake and transport across physiological barriers. Biometals.

[11] Pulgar V.M. (2018). Transcytosis to Cross the Blood Brain Barrier, New Advancements and Challenges. Front. Neurosci..

[12] Niewoehner J., Bohrmann B., Collin L., Urich E., Sade H., Maier P., Rueger P., Stracke J.O., Lau W., Tissot A.C. (2014). Increased Brain Penetration and Potency of a Therapeutic Antibody Using a Monovalent Molecular Shuttle. Neuron.

[13] Bien-Ly N., Yu J.Y., Bumbaca D., Elstrott J., Boswell C.A., Zhang Y., Luk W., Lu Y., Dennis M.S., Weimer R.M. (2014). Transferrin receptor (TfR) trafficking determines brain uptake of TfR antibody affinity variants. J. Exp. Med..

[14] Yu Y.J., Zhang Y., Kenrick M., Hoyte K., Luk W., Lu Y., Atwal J., Elliott J.M., Prabhu S., Watts R.J. (2011). Boosting brain uptake of a therapeutic antibody by reducing its affinity for a transcytosis target. Sci. Transl. Med..

[15] Villasenor R., Schilling M., Sundaresan J., Lutz Y., Collin L. (2017). Sorting Tubules Regulate Blood-Brain Barrier Transcytosis. Cell Rep..

[16] Gonzalez A.M., Leadbeater W.E., Burg M., Sims K., Terasaki T., Johanson C.E., Stopa E.G., Eliceiri B.P., Baird A. (2011). Targeting choroid plexus epithelia and ventricular ependyma for drug delivery to the central nervous system. BMC Neurosci..

[17] Rouault T.A., Zhang D.-L., Jeong S.Y. (2009). Brain iron homeostasis, the choroid plexus, and localization of iron transport proteins. Metab. Brain Dis..

[18] Abbott N.J., Pizzo M.E., Preston J.E., Janigro D., Thorne R.G. (2018). The role of brain barriers in fluid movement in the CNS: Is there a ‘glymphatic’ system?. Acta Neuropathol..

[19] Haqqani A.S., Caram-Salas N., Ding W., Brunette E., Delaney C.E., Baumann E., Boileau E., Stanimirovic D. (2013). Multiplexed evaluation of serum and CSF pharmacokinetics of brain-targeting single-domain antibodies using a NanoLC-SRM-ILIS method. Mol. Pharm..

[20] Querfurth H.W., LaFerla F.M. (2010). Alzheimer’s disease. N. Engl. J. Med..

[21] Spencer B., Masliah E. (2014). Immunotherapy for Alzheimer’s disease: Past, present and future. Front. Aging Neurosci..

[22] Sevigny J., Chiao P., Bussière T., Weinreb P.H., Williams L., Maier M., Dunstan R., Salloway S., Chen T., Ling Y. (2016). The antibody aducanumab reduces Aβ plaques in Alzheimer’s disease. Nat. Publ. Gr..

[23] Panza F., Lozupone M., Logroscino G., Imbimbo B.P. (2019). A critical appraisal of amyloid-β-targeting therapies for Alzheimer disease. Nat. Rev. Neurol..

[24] Selkoe D.J., Hardy J. (2016). The amyloid hypothesis of Alzheimer’s disease at 25 years. EMBO Mol. Med..

[25] Lindberg H., Hard T., Lofblom J., Stahl S. (2015). A truncated and dimeric format of an Affibody library on bacteria enables FACS-mediated isolation of amyloid-beta aggregation inhibitors with subnanomolar affinity. Biotechnol. J..

[26] Hoyer W., Grönwall C., Jonsson A., Ståhl S., Härd T. (2008). Stabilization of a β-hairpin in monomeric Alzheimer’s amyloid-β peptide inhibits amyloid formation. Proc. Natl. Acad. Sci. USA.

[27] Boutajangout A., Lindberg H., Awwad A., Paul A., Baitalmal R., Almokyad I., Höidén-Guthenberg I., Gunneriusson E., Frejd F.Y., Härd T. (2019). Affibody-Mediated Sequestration of Amyloid β Demonstrates Preventive Efficacy in a Transgenic Alzheimer’s Disease Mouse Model. Front. Aging Neurosci..

[28] Hultqvist G., Syvanen S., Fang X.T., Lannfelt L., Sehlin D. (2017). Bivalent Brain Shuttle Increases Antibody Uptake by Monovalent Binding to the Transferrin Receptor. Theranostics.

[29] Lee H.J., Engelhardt B., Lesley J., Bickel U., Pardridge W.M. (2000). Targeting Rat Anti-Mouse Transferrin Receptor Monoclonal Antibodies through Blood-Brain Barrier in Mouse. J. Pharmacol. Exp. Ther..

[30] Cabezón I., Manich G., Martín-Venegas R., Camins A., Pelegrí C., Vilaplana J. (2015). Trafficking of Gold Nanoparticles Coated with the 8D3 Anti-Transferrin Receptor Antibody at the Mouse Blood–Brain Barrier. Mol. Pharm..

[31] Jonsson A., Dogan J., Herne N., Abrahmsén L., Nygren P.-Å. (2008). Engineering of a femtomolar affinity binding protein to human serum albumin. Protein Eng. Des. Sel..

[32] Zorzi A., Linciano S., Angelini A. (2019). Non-covalent albumin-binding ligands for extending the circulating half-life of small biotherapeutics. MedChemComm.

[33] Kim D., Jeon H., Ahn S., Choi W.I., Kim S., Jon S. (2017). An approach for half-life extension and activity preservation of an anti-diabetic peptide drug based on genetic fusion with an albumin-binding aptide. J. Control Release.

[34] Tan H., Su W., Zhang W., Wang P., Sattler M., Zou P. (2018). Recent Advances in Half-life Extension Strategies for Therapeutic Peptides and Proteins. Curr. Pharm. Des..

[35] Sleep D., Cameron J., Evans L.R. (2013). Albumin as a versatile platform for drug half-life extension. Biochim. Biophys. Acta.

[36] Johansson M.U., Frick I.M., Nilsson H., Kraulis P.J., Hober S., Jonasson P., Linhult M., Nygren P.-Å., Uhlén M., Björck L. (2002). Structure, specificity, and mode of interaction for bacterial albumin-binding modules. J. Biol. Chem..

[37] Ståhl S., Gräslund T., Eriksson Karlström A., Frejd F.Y., Nygren P.Å., Löfblom J. (2017). Affibody Molecules in Biotechnological and Medical Applications. Trends Biotechnol..

[38] Affibody ClinicalTrials.gov Identifier: NCT03591887, A Study to Evaluate ABY-035 in Subjects With Moderate-to-severe Plaque Psoriasis (AFFIRM-35); 2018 Jul 19 (cited 2020 Feb 19). NCT03591887.

[39] Luz D., Chen G., Maranhão A.Q., Rocha L.B., Sidhu S., Piazza R.M. (2015). Development and Characterization of Recombinant Antibody Fragments That Recognize and Neutralize In Vitro Stx2 Toxin from Shiga Toxin-Producing Escherichia coli. PLoS ONE.

[40] Boado R.J., Zhang Y., Wang Y., Pardridge W.M. (2009). Engineering and expression of a chimeric transferrin receptor monoclonal antibody for blood–brain barrier delivery in the mouse. Biotechnol. Bioeng..

[41] Giannetti A.M., Snow P.M., Zak O., Björkman P.J. (2003). Mechanism for Multiple Ligand Recognition by the Human Transferrin Receptor. PLoS Biol..

[42] Yang B., Kim J.C., Seong J., Tae G., Kwon I. (2018). Comparative studies of the serum half-life extension of a protein via site-specific conjugation to a species-matched or -mismatched albumin. Biomater. Sci..

[43] Wang Q., Delva L., Weinreb P.H., Pepinsky R.B., Graham D., Veizaj E., Cheung A.E., Chen W., Nestorov I., Rohde E. (2018). Monoclonal antibody exposure in rat and cynomolgus monkey cerebrospinal fluid following systemic administration. Fluids Barriers CNS.

[44] Stocki P., Szary J.M., Jacobsen C.L., Demydchuk M., Northall L., Moos T., Walsh F.S., Rutkowski J.L. (2019). High efficiency blood-brain barrier transport using a VNAR targeting the Transferrin Receptor 1 (TfR1). bioRxiv.

[45] Liu X., Van Natta K., Yeo H., Vilenski O., Weller P.E., Worboys P.D., Monshouwer M. (2007). Unbound Drug Concentration in Brain Homogenate and Cerebral Spinal Fluid at Steady State as a Surrogate for Unbound Concentration in Brain Interstitial Fluid. Drug Metab. Dispos..

[46] Shen D.D., Artru A.A., Adkison K.K. (2004). Principles and applicability of CSF sampling for the assessment of CNS drug delivery and pharmacodynamics. Adv. Drug Deliv. Rev..

[47] Pardridge W.M. (2020). Blood-Brain Barrier and Delivery of Protein and Gene Therapeutics to Brain. Front. Aging Neurosci..

[48] Triguero D., Buciak J., Pardridge W.M. (1990). Capillary depletion method for quantification of blood-brain barrier transport of circulating peptides and plasma proteins. J. Neurochem..

[49] Bickel U. (2005). How to measure drug transport across the blood-brain barrier. NeuroRx.

[50] Jennbacken K., Wågberg F., Karlsson U., Eriksson J., Magnusson L., Chimienti M., Ricchiuto P., Bernström J., Ding M., Ross-Thriepland D. (2019). Phenotypic Screen with the Human Secretome Identifies FGF16 as Inducing Proliferation of iPSC-Derived Cardiac Progenitor Cells. Int. J. Mol. Sci..

[51] Altai M., Leitao C.D., Rinne S.S., Vorobyeva A., Atterby C., Ståhl S., Tolmachev V., Löfblom J., Orlova A. (2018). Influence of Molecular Design on the Targeting Properties of ABD-Fused Mono- and Bi-Valent Anti-HER3 Affibody Therapeutic Constructs. Cells.

