# Supplementary material for: An Affibody Molecule Is Actively Transported into the Cerebrospinal Fluid via Binding to the Transferrin Receptor
Source: Int J Mol Sci. 2020 Apr 23;21(8):2999. doi: 10.3390/ijms21082999 (PMC7215652; doi:10.3390/ijms21082999)
Supplement: Supplementary file 1 [file ijms-21-02999-s001.pdf]

Supplementary material

An affibody molecule is actively transported into the cerebrospinal fluid via binding to the transferrin receptor

Sebastian W. Meister, Linnea C. Hjelm, Melanie Dannemeyer, Hanna Tegel, Hanna Lindberg, Stefan Ståhl and John Löfblom#

Department of Protein Science, School of Engineering Sciences in Chemistry, Biotechnology and Health, KTH Royal Institute of Technology, AlbaNova University Centre, SE-106 91 Stockholm, Sweden.

#Corresponding author: John Löfblom; Telephone: +46 8 790 9659; E-mail: lofblom@kth.se

Figure S1

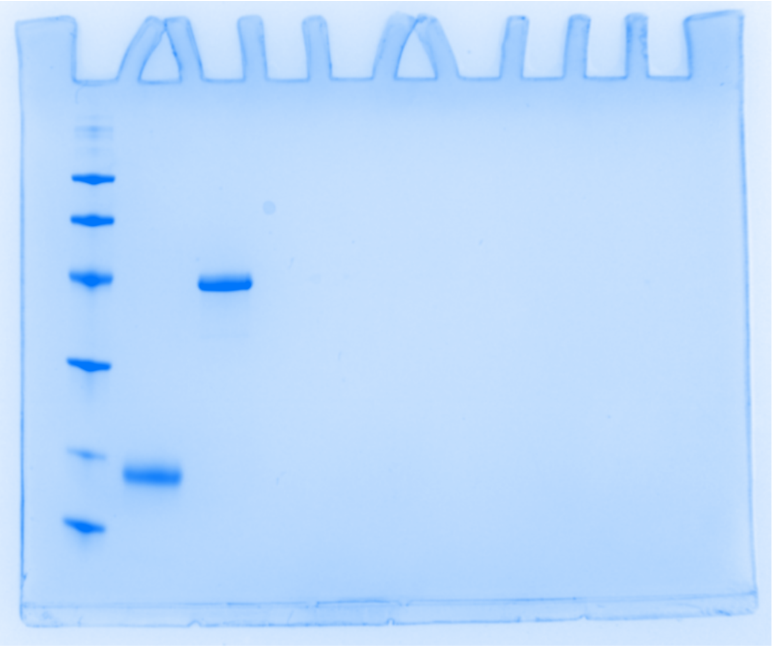

Supplementary Fig. 1. Non-cropped SDS-PAGE analysis of purified proteins. Purified scFv8D3-Z_{SYM73}-ABD and Z_{SYM73}-ABD both appear as a single band of the correct size.

Figure S2

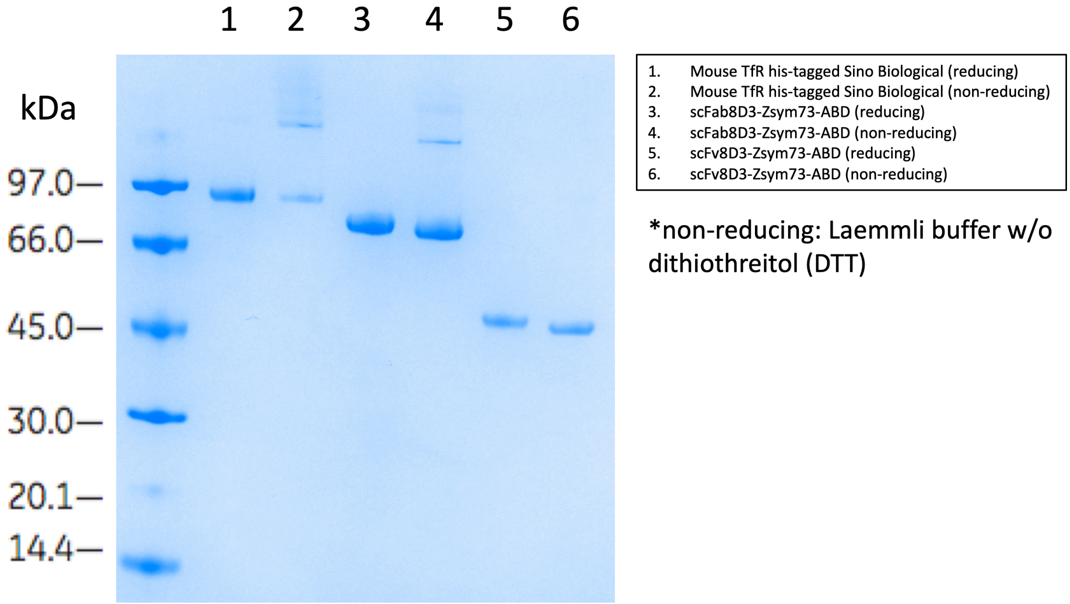

Supplementary Fig. 2. SDS-PAGE analysis of reduced (lane 1, 3 and 5) and non-reduced (lane 2, 4 and 6) recombinant proteins.

Figure S3

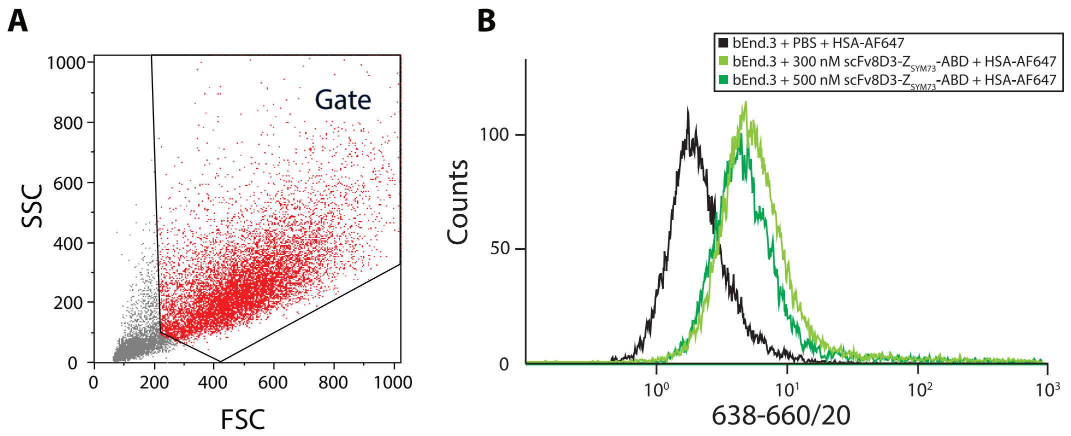

Supplementary Fig. 3. (A) Representative flow-cytometric analysis of mouse brain endothelial cells (bEnd.3) with region used for gating indicated in the dot plot. (B) Flow-cytometric analysis of scFv8D3-Z_{SYM73}-ABD binding to mouse brain endothelial cells (bEnd.3). Representative histograms showing results from the flow-cytometric analysis of bEnd.3 cells treated with 300 nM, and 500 nM scFv8D3-Z_{SYM73}-ABD. The wavelength of the excitation laser and bandwidth of the fluorescence detection filter is shown on the x-axis label in nm.

Supplementary table 1

	Time point	Animal ID	C_{serum} (nM)	C_{CSF} (nM)	C_{CSF}/ C_{serum} (%)
Z_{SYM73}-ABD	3 h	1199	1274.50	0.95	0.074
	3 h	1200	1238.07	24.00	1.939
	3 h	1201	1392.90	1.88	0.135
	3 h	1202	1207.10	1.05	0.087
	3 h	1203	1400.18	2.94	0.210
	3 h	1204	1438.43	2.04	0.142
	3 h	1205	1811.84	1.61	0.089

	Time point	Animal ID	C_{serum} (nM)	C_{CSF} (nM)	C_{CSF}/ C_{serum} (%)
Z_{SYM73}-ABD	24 h	1206	626.07	0.90	0.144
	24 h	1207	674.15	1.04	0.154
	24 h	1208	673.61	1.51	0.224
	24 h	1209	892.73	1.05	0.117
	24 h	1210	847.92	1.19	0.140
	24 h	1211	715.68	1.94	0.272
	24 h	1212	694.37	1.45	0.210

	Time point	Animal ID	C_{serum} (nM)	C_{CSF} (nM)	C_{CSF}/ C_{serum} (%)
Z_{SYM73}-ABD	48 h	1213	277.95	0.53	0.189
	48 h	1214	310.59	12.75	4.106
	48 h	1215	419.00	0.48	0.114
	48 h	1216	346.14	0.69	0.198
	48 h	1217	260.17	1.50	0.576
	48 h	1218	273.57	1.06	0.389
	48 h	1219	332.44	4.45	1.340

	Time point	Animal ID	C_{serum} (nM)	C_{CSF} (nM)	C_{CSF}/ C_{serum} (%)
scFv8D3-Z_{SYM73}-ABD	3 h	1220	868.47	6.89	0.794
	3 h	1221	1012.61	0.69	0.068
	3 h	1222			
	3 h	1223	565.17	0.47	0.083
	3 h	1224	842.94	1.08	0.128

	Time point	Animal ID	C_{serum} (nM)	C_{CSF} (nM)	C_{CSF}/ C_{serum} (%)
scFv8D3-Z_{SYM73}-ABD	24 h	1225	120.93	1.95	1.615
	24 h	1226	70.03	7.04	10.059
	24 h	1227	146.61	1.01	0.689
	24 h	1228	82.19	1.17	1.421
	24 h	1229	124.68	2.49	1.998

	Time point	Animal ID	C_{serum} (nM)	C_{CSF} (nM)	C_{CSF}/ C_{serum} (%)
scFv8D3-Z_{SYM73}-ABD	48 h	1230	3.69	n.d.	n.d.
	48 h	1231	14.50	n.d.	n.d.
	48 h	1232	28.49	0.62	2.159
	48 h	1233	19.54	7.19	36.783
	48 h	1234	39.60	0.69	1.730

Mice received a 87.8 nmol/kg dose of either Z_{SYM73}-ABD or scFv8D3-Z_{SYM73}-ABD and terminated at the indicated time point. Animal 1222 died upon injection due to an air bubble in the syringe. Orange marked CSF samples were contaminated with blood and excluded from the analysis. Grey marked CSF sampled had protein concentration below the sensitivity of the ELISA.
